# Haemolytic anaemia and hepatocitolysis associated with hypermagnesaemia by repeated exposures to copper–calcium fungicides

**DOI:** 10.1080/14756366.2017.1409745

**Published:** 2017-12-12

**Authors:** Constantin Bălăeţ, Bogdan Ioan Coculescu, Maria Bălăeţ, Gheorghe Manole, Gabi Valeriu Dincă

**Affiliations:** a Faculty of General Nursing, Bioterra University, Bucharest, Romania;; b Lil Med Clinic, Bucharest, Romania;; c Faculty of Medicine, Titu Maiorescu University, Bucharest, Romania;; d Center for Military Medical Scientific Research, Bucharest, Romania;; e Imperial College London, London, UK;; f Clinical Hospital Colentina, Bucharest, Romania

**Keywords:** Copper–calcium fungicide, hypercupraemia, oxidative stress, haemolysis, ineffective erythropoiesis, hypermagnesaemia, hepatocytic cytolysis

## Abstract

For the medical practice, our manuscript acts as a signal, despite only presenting three cases which feature the association between hepatocytolysis, haemolysis and hypermagnesaemia. This clinical–biologic triad was highlighted with the workers who through the nature of their profession were exposing themselves periodically to vapours which contained copper sulphate neutralised with calcium hydroxide, a fungicide used for fruit trees. We are exclusively assessing the haematological perturbation. In this aetiological context, the generating mechanism for haemolysis is very probable biochemical, where hypercupraemia interferes with cellular antioxidant defence mechanisms. Hypothetically, the role of the redox homeostasis disorder in the intravascular destruction of erythrocytes is sustained, and particularly the coexistence of cell cytolysis in the medullary erythroid compartment, which can be assimilated with a possible ineffective erythropoiesis.

## Introduction

(a) Physiologically, *the copper* constituent of over 50 of the body's enzymes (cytochrome c oxidase, superoxide dismutase, ferroxidases, monoamine oxidase, β-monooxygenase dopamine etc.) participates in the synthesis of collagen, macroelectronic compounds, in the cross-linking of collagen fibres, elastin and keratin, in haematopoiesis, immunity, neural tissue development, pigmentation of the skin, deposition of calcium in the bones and it also works as a reducing agent of the concentration of reactive oxidative species (ROS)^1^. The fulfilment of last function by Cu^2+^ is the expression of has ability to accept or donate electrons, but also of its intervention as a coenzyme in the synthesis of various major antioxidant enzymes or enzymes involved in energy generation^2^^,^^3^. For the development of these roles, it is compulsory to maintain Cu^2+^ homoeostasis, as its dysregulation is disease-causing. The presence of this ion in high concentrations induces hepatic, renal and neuronal lesions, anaemia, coagulopathy, immunodepression, etc^4^.

**Figure 1.**
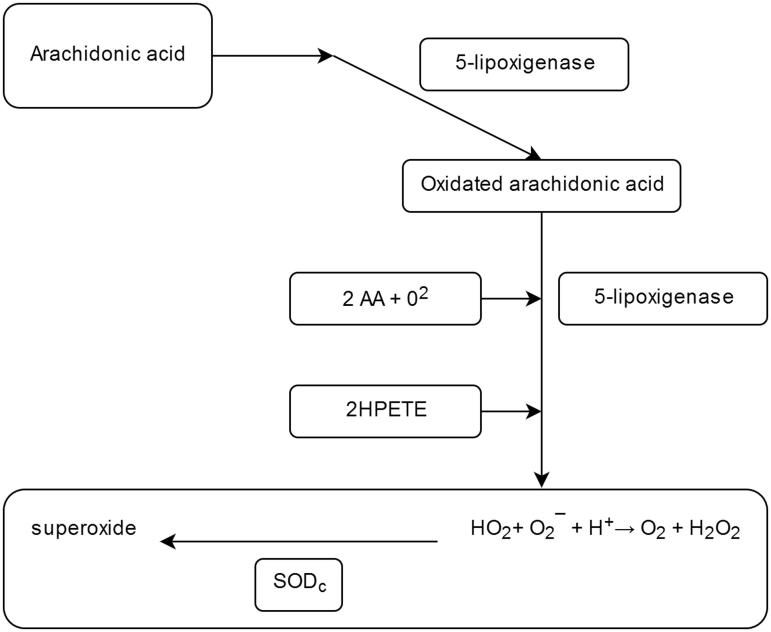
Production of ROS – the role of superoxide dismutase (SODc) as an antioxidant agent^17^.

Pathogenically, many of these effects induced by excess Cu^2+^ can be attributed, even partially, to the oxidative effect happening at membrane or macromollecular level in various cells. The focus on this ion as a cause of disease is based on its ability to produce large quantities of reactive oxygen species, thus installing the state of regional oxidative stress^2^ (Figure 1).

An important role in the regeneration and in the defence of cells against oxidative stress is attributed to the superoxide-dismutase (SOD). At cellular level, the three representatives of this family of metalo-enzymes involved in these aerobic cellular processes are ubiquitously distributed (Table 1).

Physiologically, the SOD antioxidant action consists in the dismutation of the superoxidant anion which was generated from the cellular level activity, thus generating an oxygen molecule and a peroxide molecule which is inactivated by catalase, due to these two enzymes having a synergistic action:

$${\text{2O}}_{\text{2}}^{-}+{\text{2H}}^{+}\to {\text{O}}_{\text{2}}+{\text{H}}_{\text{2}}{\text{O}}_{\text{2}}$$

Catalytically, the reaction proceeds at a very high velocity (dissociation constant, *k* = 1.6–1.8 × 10^9^ M^−1^·s^−1^), compared with that of the non-enzymatic pathway (9.7 × 10^7^ M^−1 ^s^−1^)^1^^,^^5^.

(b) Optimal cell function and understanding the pathogenicity of various diseases is also conditioned by the cytosolic concentration of *magnesium* ion. This, by being an intermediate in over 300 reactions, plays an important role as a cellular biocatalyst in maintaining a biochemical equilibrium. As a secondary messenger, magnesium is involved in cell growth and modulation of apoptosis, by participating in the synthesis of nucleic acids and proteins. However, the regulation of intracellular Mg^2+^ concentration is complex, involving:

- transient receptor potential melastin 6 and 7 (TRPM6 and TRPM7);
- solute carrier family 41 (SLC41A1/A2), where the SLC41A1 isomembrane is involved in the Na^+^/Mg^2+^ transmembrane transport; and
- magnesium transfer protein (MgT1)^6^.

## Materials and methods

In the spring of 2016, three male patients belonging to the same family: father (50 years old) and sons (27 years and 29 years old) presented themselves to the occupational medicine physician of the Lil Med Clinic Bucharest for the following symptomatology: dry cough, intermittent and recurrent balance disorder, subfebrile condition, myalgia, asthenia, metallic taste, quasipermanent nausea, vomiting, pelvic limb paresthesia, pain in the right hipochondiral region. The anamnesis revealed that the patients had been working for a few years in fruit growing, participating, among other things, in the treatment of fruit trees with cupro-calcic fungicide, called bordehyde/copper sulphate neutralised with calcium hydroxide. The fungicides utilised varied with the vegetation periods: when no vegetation was present the concentration of the solution was 1–2%, and when vegetation was present it was 0.50–0.75%. Three different fungicides from three different brands were utilised. Although frequent tree spraying was supposed to be carried out in days with no wind, the impossibility of preserving the efficiency of the mixture for the day, required continued action under unfavourable atmospheric conditions, such as the presence of wind. The workers reported that they did not respect the labour safety rules, so the atmospheric factor constituted a favouring factor for oral and respiratory exposure to the fine copper particles dissolved in the solution. Regardless of the penetration pathway, diffusion throughout the body occurs in the same way: as cupric albumin.

The study was conducted in accordance with the Declaration of Helsinki.

In all patients, the general clinical examination revealed the presence of cold skin, pale mucous membranes, orthostatic hypotension and grade-1 hepatomegaly, as evidenced by abdominal ultrasound.

The biological investigation of the patients consisted in venous blood tests:

(a) Routine examinations mandatory for each patient: – haematological: haemoleucogram (CBC), haemoglobin (Hb), haematocrit (Ht), ESR, – and biochemistry: glucose, bilirubin (direct, indirect, total), transaminases (TGP, TGO), alkaline phosphatase (ALP), creatinine, cholesterol high-density lipids (HDL), cholesterol low-density lipids (LDL), triglycerides, C-reactive protein (CRP), ceruloplasmin, calcium, potassium, sodium.

(b) Targeted investigations: cupraemia and magnesaemia. The venous blood necessary to determine the copper has been harvested using as an anticoagulant the lithium heparinate, and the venous blood necessary to determine the magnesium ions has been harvested using a separating gel. In order to avoid alteration of the magnesium level results, the blood was taken when the patients were laying down, in order to avoid venous stasis with the elastic band; the blood was taken only after washing the talc gloves, since they may contain magnesium which could influence the result. Copper was determined by atomic absorption spectrometry.

(c) To determine the cupriuria and the urobilinogenuria, the 24 h urine has been harvested, using a preservative. During the collection, the established requirements were met, such as the exclusion of the first urine from the beginning of the procedure, not touching the interior of the container, proper hydration and the storage of urine collected in the refrigerator. Because there are diurnal variations in urobilinogen urinary excretion, a sample of total urine was used and the photometric method provided qualitative information on this pigment metabolite.

## Results

The results of the laboratory investigations are centralised in Table 2.

**Table 1.** The distribution and role of the isoenzymes/isoforms SOD at cellular level.

Type and structure of the enzyme	Abbreviation	Localisation		Antioxidant action/neutralisation of ROS
SOD copper-zinc (dimeric)	Cu–Zn (SOD_1_)	All types of cells	cytoplasm	By activating the endoplasmic reticulum or other cellular organelles, except for mitochondria
SOD-mangan (tetrameric)	MnSOD (SOD_2_)		mitochondria matrix	The process of synthesising macroergic compounds, through electron transport chains
SOD–iron or nikel	ECSOD (SOD_3_)	All vascular endothelium and lungs	cell’s membrane	From the activity at cellular membrane level

**Table 2.** Results of laboratory tests for the three patients in the study.

	Reference values			Patients		
Parameters	Measure units		References (normal)	T.V (50 years old)	T.M. (29 years old)	T.G. (27 years old)
Hb	g/dL		13–16	12	12.3	12.8
ESR (men)	mm/1 h		<20	30	20	28
Blood glucose	mg/dL		73–110	105	95	85
Bilirubin						
total	mg/dL		<1.2	1.5	1.7	1.6
unconjugated	mg/dL		<0.8	1.1	1.3	1.2
TGP (ALT)	I.U./L		5–21	70	40	45
TGO (AST)	I.U./L		5–40	30	30	30
ALP	U/L		30–126	140	135	125
creatinine	mg/%ml		<1.2	0.9	1	0.85
HDL cholesterol	mg/dL		≥40	45	44	40
LDL cholesterol	mg/dL		<130	120	105	90
Triglycerides	mg/%ml		30–135	280	200	170
CRP	mg/dL		<0.5	1	0.8	1.5
Free serum copper	µg %ml		70–152	360	300	290
Serum ceruloplasmin	mg/%ml		15–30	40	38	41
Electrolytes						
Mg^2+^	mg%dL		1.6–2.55	5.4	5.9	5.7
	critical level: ≥4,5 mg/dl					
Ca^2+^ total	mg%dL		8.4–10.3	8.5	7.7	7.9
Na^+^	mmol/L		135–145	137	135	138
K^+^	mmol/L		3.5–5	3.3	3.8	4

Urinary copper	mg/24 h		10–60	90	80	85
Urinary urobilinogen	photometry		negative	positive	positive	positive

## Discussions

(a) Cu^2+^ becomes harmful to the human body only at high levels in the blood, which are also present at tissue level. The pathogenic effects induced by excess Cu^2+^ are stronger if the ion is not coupled to the carrier. The oxidative action it induces leads to cellular functional disruption of most tissues: haematopoietic, digestive, hepatic, etc. The ability of excess Cu^2+^ to induce acute kidney failure through developing an interstitial nephrite, shock or coma and even death is exemplified in our previous studies^1^^,^^7^^,^^8^. Recent research has shown that immune system and antioxidant enzyme degradation occurs at under 7.800 μg Cu^2+^ daily exposure levels, but it is not capable of inducing hepatocytolysis^9^. From the clinico-biological triad present in the three patients, we decided to discuss the toxic hepatocytolisis in another paper, because its mechanism is much more complex. It has a double aetiology: the regular consumption of alcohol (disclosed by the patients) and the highlighted hypercupremia. However, we are reluctant to name the hepatic cytolysis responsible for the excessive tissue copper. This is because the scientific literature does not note this, and because it is necessary for a serum concentration to be much higher for cupremia and for inducing hepatic cytolysis. On the other hand, since the scientific literature only notes that haemolysis can appear in the acute alcoholism (Zieve’s syndrome), highlighting this in the three cases can be causally attributed exclusively to hypercupremia. The decision to communicate our findings is also justified by the numerous unknown factors related to the toxic effects of Cu^2+^. Following this point, we are signalling the development of haemolysis, in all three cases, which we believe is predominantly due to inefficient erythropoiesis.

Based on the anamnesis, the clinical examination and the results of the main parameters determined, the diagnosis of chronic copper intoxication was made. The accumulation of copper in the tissues is the result of repeated exposure for a few days every two or three months to copper–calcium fungicides, which were used at work, fungicides being used four times per year. In all three cases, although there was no anaemia, only subnormal circulating haemoglobin values, there was constant haemolysis. The absence of anaemia is interpreted as the ability of the haematopoietic red marrow to compensate for haemolysis, as demonstrated by the fact that in the case of reducing the life of erythrocytes, erythropoietal activity may increase 6–8 times^2^. However, this does not exclude the possibility that the haemolytic toxic action of Cu^2+^ has been induced through the ineffective erythropoiesis. In all three cases, the existence of haemolysis is supported by the presence of:

- high level of total bilirubin, as a result of high levels of unconjugated bilirubin. Although not very high, the indirect bilirubin level was in all cases above three-fourth of the total bilirubin value (Table 2). The “share” of hepatopathy induced by excess copper ion, which most likely also contributes to the high level of indirect bilirubinaemia identified, is the theme of a future work that we will publish. We mention that hypothetically the action mechanism we support is very likely a Cu^2+^ interference with uridine-diphospho-glucuronide-transferase enzyme activity.
- the positive photometric test, which indirectly indicates the existence of higher values of urobilinogen in the urine, even if macroscopically urine was normocrome.

The correlation of these values with those of copper shows the existence of a concordance. In all three cases, the copper levels were 17–27% higher than the normal blood levels accepted by the medical literature (Table 2).

**Figure 2.**
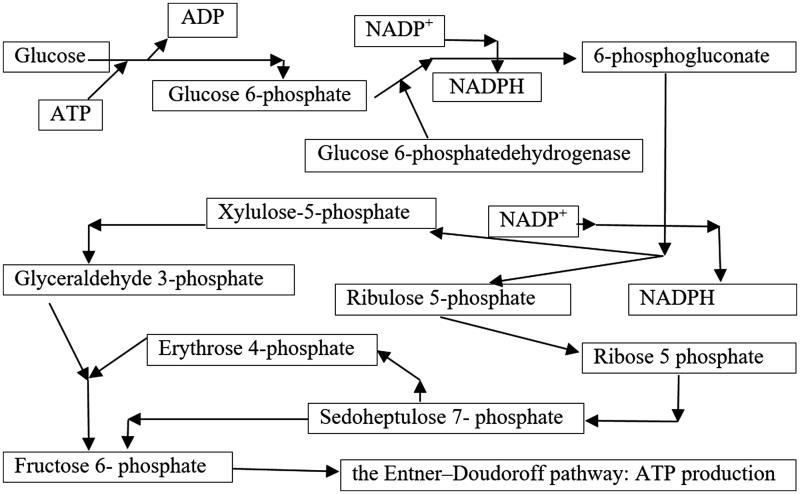
The role of pentoses shunt at the erythrocyte level in the synthesis of macro-active compounds and in the prevention of oxidation processes.

We explain the haemolytic action of excess Cu^2+^ by its oxidative capacity to interfere with pentose shunt which results in reduced synthesis of reduced glutathione. The toxic action of copper ion is exerted on the glucose-6-phosphate-dehydrogenase enzyme (G-6-PD) which catalyses the first step of the pentoses shunt deployment (Figure 2).

The reduction of intracellular glutathione-disulphide reductase (GSR) has as a direct consequence on the reduction of peroxidation prevention of red cell membrane lipids, because G-6-PD provides the potential reduction in nicotinadenyl nucleotide-phosphate (NADP^+^), a compound which protects the functional structures of erythrocytes from oxidation processes. This enzyme reduces oxidised glutathione disulphide (GSSG) to the sulphhydryl form GSH, which is an important cellular antioxidant^10^:

$$\text{GSSG}+\text{NADPH}+{\text{H}}^{+}\overset{\text{glutathione}-\text{disufide reductase}}{⇄}\text{2 GSH}+{\text{NADP}}^{+}$$

At the stroma level of red blood cells, diminishing the NADPH synthesis capacity leads to insufficient presence of GSH, which allows not only glutathione disulphide (GSSH) formation but also the oxidation of proteins at this level or from the erythrocyte membrane constitution^11^^,^^12^. Thus, methemoglobinis formed in the intra-cytoplasmic erythrocyte space. It forms in the form of aggregates on the internal surface of the membrane of the blood, stiffening it and deforming it. Morphological deformations induce haemolysis in the sarcoplasmic reticulum of the spine. Oxidative degradation processes may also occur in the structural proteins or enzymes contained in the erythrocyte membrane, also leading to haemolysis^13^. Based on literature data, the G-6-PD deficiency is determined by one of the anomalies that may affect the gene involved in the synthesis of this enzyme (small deletions or point mutations), with the following consequences:

- reducing the amount of normal enzymes produced at the ribosomal level;
- qualitative disorder, in the sense of diminishing the enzymatic affinity for the specific substrate;
- low stability or even instability of the enzymatic molecule;
- synthesis of molecules with associated alterations^14^.

Considering the absence of the nucleus at the level of the haematite, we estimate that the genetic abnormality is induced by the copper ion in the red series cells in the differentiation and maturation compartment of the red marrow, in order to be able to interfere with the protein synthesis and the action of cross-linking ion. Such a hypothesis would also explain the particularity of the three cases of featuring lower limit values of haemoglobin, with erythrocytes of normocromial type and normal haematic volume, but with three parameters indicating the presence of haemolysis. Our supposition is not identical to the hypothesis advocated by Uriu-Adams and Keen who localise haemolysis intravascularily^3^. In our hypothesis, the haemolysis site is especially at the medial erythroid compartment level, which is pathogenic and features an ineffective erythropoiesis. Useful for our hypothesis would have been to perform a biopsy/medulogram that would have revealed the existence, or absence, of a reactive hepatocellular hyperplasia. We did not decide to pursue it because of the inexistence of a severe anaemia, where further harm to the patient could not justify scientific interest.

Medical literature reports that a particularity of Cu^2+^-induced haemolysis may occur in severe forms associated with coma and in conditions featuring a coexistence with normal copper^15^^,^^16^.

**Figure 3.**
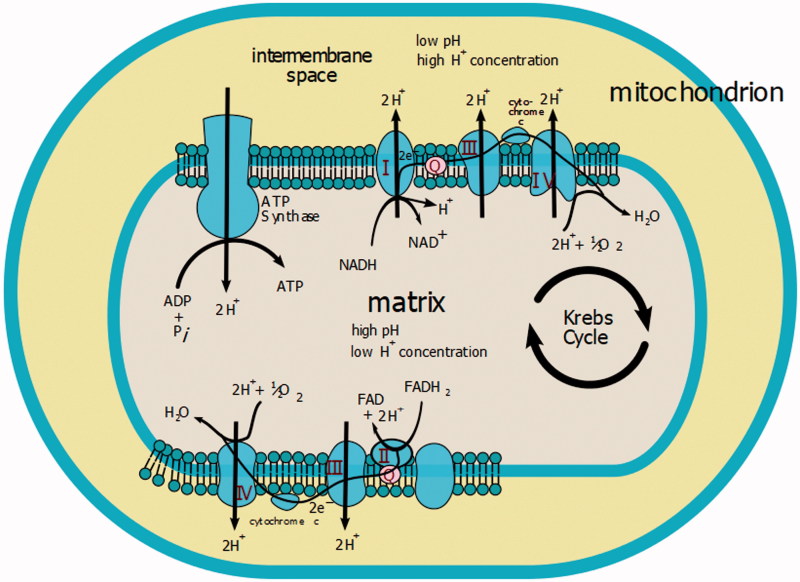
Involvement of cytochrome oxidase in the synthesis of macro-active compounds^18^.

In our hypothesis, another pathogenic mechanism responsible for installing haemolysis in such cases is also the effect of intracellular energy production, via copper enzyme, cytochrome-c-oxidase. The role of the catalyst enzyme is reducing molecular oxygen in water, during which time the enzyme transfers two hydrogen atoms into the mitochondrial intermembrane space, thus producing an electric gradient used by the mitochondria to synthesise the ATP molecule (Figure 3).

(b) Similar to copper, *magnesium* is another microelement, defined as though it is found in the body in a small proportion (0.05% of the total body weight), intervenes structurally and functionally at a cellular level. Although it is predominantly distributed in bone (over 70% of the total amount of magnesium in the human body – about 14 g), 1% is in plasma and another is about 5.2 mEq/L in erythrocytes. Inside cells Mg^2+^ is distributed in the mitochondria and in the nucleus. Mg^2+^ is an activator of over 300 enzymes involved in energy synthesis, especially from carbohydrates and from proteins and nucleic acids.

In the three cases studied by us, characterised by the presence of a frustum, normochemical normocromes, but ethiopathogenic haemolytic, the determinations highlighted the presence of an increase in magnesaemia, which we interpret as the expression of an increased release of ions from haemolytic erythrocytes under copper intoxication. In particular, we have found that the higher the level of serum copper, the higher the haemolysis is assessed by the concentration of indirect bilirubin, and as a consequence that of magnesium. In all three cases the magnesium value was above the 50% higher critical level, and twice above the normal reference value admitted by the literature, as shown in Table 2.

## Conclusions

Although it only discusses three cases of cuprous intoxication, and most likely that the clinical form is of a chronic type, the observations indicate the possibility of the haemolytic anaemia associated with an increase in circulating magnesium. In all cases, a correlation between the biological haemolysis signalling tests and the level of copper and magnesium could be established. Pathogenetically, the mechanisms that generate these clinical–biological changes can only be explained by the role of excessive copper ion inducing oxidative stress and haemolysis.

In our hypothesis, its location is both intravascular, but especially at the level of the red marrow, which is equivalent to the process of ineffective erythropoiesis. Additionally, we admit that in the induction of haemolysis the existence of a deficiency of erythrocytic energy participates associatively by disrupting the catabolism of glucose as a result of the interference of the pentose-phosphate shunt.
